# Risk Factors for Death among Children Less than 5 Years Old Hospitalized with Diarrhea in Rural Western Kenya, 2005–2007: A Cohort Study

**DOI:** 10.1371/journal.pmed.1001256

**Published:** 2012-07-03

**Authors:** Ciara E. O'Reilly, Peter Jaron, Benjamin Ochieng, Amek Nyaguara, Jacqueline E. Tate, Michele B. Parsons, Cheryl A. Bopp, Kara A. Williams, Jan Vinjé, Elizabeth Blanton, Kathleen A. Wannemuehler, John Vulule, Kayla F. Laserson, Robert F. Breiman, Daniel R. Feikin, Marc-Alain Widdowson, Eric Mintz

**Affiliations:** 1 Division of Foodborne, Waterborne, and Environmental Diseases, National Center for Emerging and Zoonotic Infectious Diseases, Centers for Disease Control and Prevention, Atlanta, Georgia, United States of America; 2 Kenya Medical Research Institute/Centers for Disease Control and Prevention, Kisumu, Kenya; 3 Division of Viral Diseases, National Center for Immunization, and Respiratory Diseases, Centers for Disease Control and Prevention, Atlanta, Georgia, United States of America; 4 Centre for Global Health Research, Kenya Medical Research Institute, Kisumu, Kenya; 5 Center for Global Health, Centers for Disease Control and Prevention, Atlanta, Georgia, United States of America; World Health Organization, Switzerland

## Abstract

**Background:**

Diarrhea is a leading cause of childhood morbidity and mortality in sub-Saharan Africa. Data on risk factors for mortality are limited. We conducted hospital-based surveillance to characterize the etiology of diarrhea and identify risk factors for death among children hospitalized with diarrhea in rural western Kenya.

**Methods and Findings:**

We enrolled all children <5 years old, hospitalized with diarrhea (≥3 loose stools in 24 hours) at two district hospitals in Nyanza Province, western Kenya. Clinical and demographic information was collected. Stool specimens were tested for bacterial and viral pathogens. Bivariate and multivariable logistic regression analyses were carried out to identify risk factors for death. From May 23, 2005 to May 22, 2007, 1,146 children <5 years old were enrolled; 107 (9%) children died during hospitalization. Nontyphoidal *Salmonella* were identified in 10% (118), *Campylobacter* in 5% (57), and *Shigella* in 4% (42) of 1,137 stool samples; rotavirus was detected in 19% (196) of 1,021 stool samples. Among stools from children who died, nontyphoidal *Salmonella* were detected in 22%, *Shigella* in 11%, rotavirus in 9%, *Campylobacter* in 5%, and *S*. Typhi in <1%. In multivariable analysis, infants who died were more likely to have nontyphoidal *Salmonella* (adjusted odds ratio [aOR] = 6·8; 95% CI 3·1–14·9), and children <5 years to have *Shigella* (aOR = 5·5; 95% CI 2·2–14·0) identified than children who survived. Children who died were less likely to be infected with rotavirus (OR = 0·4; 95% CI 0·2–0·8). Further risk factors for death included being malnourished (aOR = 4·2; 95% CI 2·1–8·7); having oral thrush on physical exam (aOR = 2·3; 95% CI 1·4–3·8); having previously sought care at a hospital for the illness (aOR = 2·2; 95% CI 1·2–3·8); and being dehydrated as diagnosed at discharge/death (aOR = 2·5; 95% CI 1·5–4·1). A clinical diagnosis of malaria, and malaria parasites seen on blood smear, were not associated with increased risk of death. This study only captured in-hospital childhood deaths, and likely missed a substantial number of additional deaths that occurred at home.

**Conclusion:**

Nontyphoidal *Salmonella* and *Shigella* are associated with mortality among rural Kenyan children with diarrhea who access a hospital. Improved prevention and treatment of diarrheal disease is necessary. Enhanced surveillance and simplified laboratory diagnostics in Africa may assist clinicians in appropriately treating potentially fatal diarrheal illness.

*Please see later in the article for the Editors' Summary*

## Abstract 2

A hospital-based surveillance study conducted by Ciara O'Reilly and colleagues describes the risk factors for death amongst children who have been hospitalized with diarrhea in rural Kenya.

## Editors' Summary

**Background:**

Diarrhea—passing three or more loose or liquid stools per day—kills about 1.5 million young children every year, mainly in low- and middle-income countries. Globally, it is the second leading cause of death in under-5-year olds, causing nearly one in five child deaths. Diarrhea, which can lead to life-threatening dehydration, is a common symptom of gastrointestinal infections. The pathogens (viruses, bacteria, and parasites) that cause diarrhea spread through contaminated food or drinking water, and from person to person through poor hygiene and inadequate sanitation (unsafe disposal of human excreta). Interventions that prevent diarrhea include improvements in water supplies, sanitation and hygiene, the promotion of breast feeding, and vaccination against rotavirus (a major viral cause of diarrhea). Treatments for diarrhea include oral rehydration salts, which prevent and treat dehydration, zinc supplementation, which decreases the severity and duration of diarrhea, and the use of appropriate antibiotics when indicated for severe bacterial diarrhea.

**Why Was This Study Done?:**

Nearly half of deaths from diarrhea among young children occur in Africa where diarrhea is the single largest cause of death among under 5-year-olds and a major cause of childhood illness. Unfortunately, although some of the risk factors for death from diarrhea in children in sub-Saharan Africa have been identified (for example, having other illnesses, poor nutrition, and not being breastfed), little is known about the relative contributions of different diarrhea-causing pathogens to diarrheal deaths. Clinicians need to know which of these pathogens are most likely to cause death in children so that they can manage their patients appropriately. In this cohort study, the researchers characterize the causes and risk factors associated with death among young children hospitalized for diarrhea in Nyanza Province, western Kenya, an area where most households have no access to safe drinking water and a quarter lack latrines. In a cohort study, a group of people with a specific condition is observed to identify which factors lead to different outcomes.

**What Did the Researchers Do and Find?:**

The researchers enrolled all the children under 5 years old who were hospitalized over a two-year period for diarrhea at two district hospitals in Nyanza Province, tested their stool samples for diarrhea-causing viral and bacterial pathogens, and recorded which patients died in-hospital. They then used multivariable regression analysis (a statistical method) to determine which risk factors and diarrheal pathogens were associated with death among the children. During the study, 1,146 children were hospitalized, 107 of whom died in the hospital. 10% of all the stool samples contained nontyphoidal *Salmonella*, 4% contained *Shigella* (two types of diarrhea-causing bacteria), and 19% contained rotavirus. By contrast, 22% of the samples taken from children who died contained nontyphoidal *Salmonella*, 11% contained *Shigella*, 9% contained rotavirus, and 5% contained *Campylobacter* (another bacterial pathogen that causes diarrhea). Compared to survivors, infants (children under 1 year of age) who died were nearly seven times more likely to have nontyphoidal *Salmonella* in their stools and children under 5 years old who died were five and half times more likely to have *Shigella* in their stools but less likely to have rotavirus in their stools. Other factors associated with death included being malnourished, having oral thrush (a fungal infection of the mouth), having previously sought hospital care for diarrhea, and being dehydrated.

**What Do These Findings Mean?:**

These findings indicate that, among young children admitted to the hospital in western Kenya with diarrhea, infections with nontyphoidal *Salmonella* and with *Shigella* (but not with rotavirus) were associated with an increased risk of death. Because this study only captured deaths in hospital and most diarrheal deaths in developing countries occur at home, these results may not accurately reflect the pathogens associated with overall childhood diarrheal deaths. In addition, they may not be generalizable to other geographical regions. Nevertheless, given that that there are currently no vaccines available for most bacterial diarrheal diseases, these findings highlight the importance of Kenya and other developing countries implementing effective strategies for the prevention and management of diarrheal diseases in children such as increasing access to improved water, sanitation, and hygiene, and community-level promotion of the use of oral rehydration solution and zinc supplements. They also suggest that enhanced surveillance and simplified laboratory diagnostics for diarrheal pathogens could help clinicians identify those children presenting to hospital with diarrhea who are at high risk of death and prioritize their treatment.

**Additional Information:**

Please access these Web sites via the online version of this summary at http://dx.doi.org/10.1371/journal.pmed.1001256.

- The World Health Organization provides information on diarrhea (in several languages); its 2009 report with UNICEF Diarrhea: why children are still dying and what can be done, which includes the WHO/UNICEF recommendations for the treatment and prevention of diarrhea in children, can be downloaded from the Internet
- The children's charity UNICEF, which protects the rights of children and young people around the world, provides information on diarrhea (in several languages)

## Introduction

Diarrhea is a major cause of morbidity and mortality among children <5 y old in sub-Saharan Africa [1],[2]. Of the estimated 4·2 million deaths in children <5 y old in Africa in 2008, diarrhea caused the largest proportion (19%); followed by pneumonia (18%), and malaria (16%) [3]. The number of childhood deaths only decreased by 4% in Africa from 2000–2003 to 2008, suggesting that interventions for these diseases are lacking in Africa [3],[4].

New tools to combat severe illness and death from rotavirus gastroenteritis have been developed [5]–[7]; recently, the World Health Organization (WHO) recommended the use of rotavirus vaccines worldwide [8]. However, besides the WHO recommendation in 2004 to use zinc for the treatment of diarrhea in children [9], limited progress has been made in the development of new effective prevention and treatment measures for other diarrheal diseases, even as availability and use has declined for the most practical interventions to prevent mortality, oral rehydration solution (ORS) and appropriate infant feeding practices during diarrheal illness [10]–[12]. Understanding the frequency, and relative severity, of diarrheal pathogens in children in sub-Saharan Africa, as well as the antimicrobial susceptibilities of bacterial diarrheal pathogens is critical to better tailor treatment regimens and stimulate new approaches for the prevention of childhood diarrhea.

Detailed information on the etiology and risk factors for fatal childhood diarrhea in sub-Saharan Africa is sparse [13]. Previous mortality studies among young children in various settings in Africa have identified young age, co-morbidity, poor nutritional status, dehydration, lack of breastfeeding, thrush, immunosuppression, prolonged diarrheal duration before hospital admission, extended hospitalization, previous hospital discharges, inter alia, as risk factors for death [14]–[20]. None of these studies identified diarrheal etiologies. Due to the scarcity of data on pathogen-specific childhood diarrheal deaths in Africa, estimates of the relative contribution of infectious diarrheal etiologies to mortality are unavailable [13].

To address the lack of data examining a range of specific infectious agents and risk factors for childhood diarrheal mortality, we carried out a cohort study among children <5 y old in western Kenya to characterize the etiologies and risk factors associated with death during hospitalization for diarrhea.

## Materials and Methods

### Setting

Bondo and Siaya District Hospitals are two large government-operated district hospitals in Nyanza Province, western Kenya; together they serve mainly a rural population of ∼750,000 people [21]. In 2003, 88% of households in this region lacked access to safe potable water, and 26% lacked latrines [22]. Nyanza Province has one of the nation's lowest immunization rates, the highest infant (125 per 1,000 live births) and child (227 per 1,000 live births) mortality rates, and the highest reported HIV prevalence (15% among persons aged 15–49 y); malaria infection and malnutrition are common [22],[23].

### Hospital-Based Surveillance

Data were prospectively collected on admission and at death or discharge for all children <5 y old hospitalized with diarrhea at Bondo and Siaya District Hospitals from May 23, 2005 to May 22, 2007. Diarrhea was defined as ≥3 loose stools within 24 h occurring in the previous 5 d. Bloody diarrhea was defined as the presence of visible blood in stool. All enrolled children were assessed clinically and treated according to the Kenya Ministry of Health (MoH) pediatric standard of care by MoH clinicians not affiliated with the study.

After explaining the study and obtaining written informed consent, trained study staff interviewed caretakers in Dholuo using a standardized supplemental diarrheal questionnaire, collecting information about patient demographics, and the child's clinical diarrheal history; caretakers' written informed consent was also sought to collect, store, and test a stool specimen from their child. The enrolled child's diarrheal treatment and outcome were derived from standardized interviews with the attending clinician, and from medical record abstraction. All reference to children who died or survived specifically relates to in-hospital death or survival. For children with multiple diarrhea admissions, only the last diarrheal episode for which the child sought care was included. In addition to the supplemental diarrhea specific questionnaire, information was collected via trained study staff of the Kenya Medical Research Institute (KEMRI)/Centers for Disease Control and Prevention (CDC) Health and Demographic Surveillance System (HDSS) inpatient surveillance program. The HDSS staff captured routine data on the enrolled child's overall clinical course, exam by the attending physician, anthropometric measurements, health-seeking behavior, treatments before the hospital visit, and laboratory findings, including malaria status. From January 1 to May 22, 2007, HDSS data were not collected at Bondo District Hospital. All enrolled children presenting with diarrhea had the supplemental diarrhea questionnaire administered; additional HDSS data were available for enrolled children who resided in the HDSS study area.

The admission diagnosis was captured by a physician on physical examination of the child at the point of initial admission to the hospital. At the time of discharge or death the physician reviewed the patient records and indicated the diagnosis on discharge/death considering all additional information available during the hospitalization, for example results from diagnostic testing, etc. We defined dehydration on physical exam as sunken eyes, loss of skin turgor, or sunken fontenelle. Fast breathing was defined as ≥60 breaths per minute for children <2 mo old, ≥50 breaths per minute for children 2–11 mo and ≥40 breaths per minute for children 12–59 mo old [24]. The presence and density of malaria parasites was determined by blood smear. After assessment of the median parasite density, high parasite density was defined as ≥20,000 parasites per µl of blood. Severe anemia was defined as a hemoglobin concentration <5 g/dl. HIV counseling and testing were not routinely offered at the time of the study; therefore HIV testing results are not available for participants. For study purposes, classification of HIV/AIDS was based on a clinical diagnosis of “immune suppression syndrome,” or documented antiretroviral therapy on medical records.

Malnutrition was defined as a z-score of <−2 for weight-for-length/height, length/height-for-age, or weight-for-age. Z-scores were calculated using a WHO SAS macro and the WHO Child Growth Standards as the reference population [25]. A restricted analysis was carried out with missing and biologically implausible values excluded [25],[26].

### Laboratory Methods

Whole stool specimens and/or rectal swabs placed in Cary-Blair transport medium were cultured for *Salmonella*, *Shigella*, *Campylobacter*, and *Vibrio* species by standard techniques, and tested for rotavirus by enzyme-linked immunoassay (EIA) (catalogue number 696004, Meridian Biosciences, Inc.) at the KEMRI/CDC laboratory in Kisumu, Kenya. *Campylobacter* isolates were tested for hippurate hydrolysis to identify *C. jejuni* (hippurate positive) from *C. coli* (hippurate negative and grew on *Campylobacter* selective media at 42°C) or *Campylobacter* species. *Vibrio cholerae* isolates were assessed for the O1 serogroup using commercial antisera (catalogue number LL-13916, Lee Labs, Becton-Dickinson) [27]. Antimicrobial susceptibilities of *Salmonella*, *Shigella*, and *Vibrio* isolates to a panel of antimicrobial agents (VWR International) (amoxicillin-clavulanic acid; ampicillin; ceftriaxone; chloramphenicol; ciprofloxacin; gentamicin; kanamycin; nalidixic acid; streptomycin; sulfisoxazole; tetracycline; trimethoprim-sulfamethoxazole) was determined by the disk diffusion method at the KEMRI/CDC laboratory in Kisumu, Kenya [28]. Isolates with moderate or intermediate susceptibility were classified as susceptible. Laboratory results were communicated to each hospital.

The CDC laboratories in Atlanta serotyped nontyphoidal *Salmonella* and *Shigella* isolates (catalogue number 294821, Denka Seiken Co. LTD), and tested *V. cholerae* isolates by PCR for cholera toxin (ctxA) [29], biotype (tcpA) [30], and species-specific gene sequences [31],[32]. Specimens EIA-positive for rotavirus from children who died were subtyped using semi-nested reverse-transcription PCR (RT-PCR) targeting two outer capsid protein genes, VP7 (G-genotype) and VP4 (P-genotype) at the KEMRI laboratories in Nairobi, Kenya [33].

Stool specimens from all 107 children who died, and because of cost and logistical constraints, specimens from a subset of 107 children who survived were tested at the CDC laboratories in Atlanta, GA by multiplex PCR for the genes of enteroaggregative *Escherichia coli* (EAEC) [34], enteropathogenic *E. coli* (EPEC) [35],[36], enterotoxigenic *E. coli* (ETEC) [37], enteroinvasive *E. coli* (EIEC) [38], Shiga toxin-producing *E. coli* (STEC) [36], and for norovirus, sapovirus, and astrovirus by real-time TaqMan RT-PCR [39],[40].

### Data Management

Data were recorded on optical character recognition enabled forms, scanned into a database using Teleform version 9 software (Verity, Inc., 2003), and subjected to validity checks.

### Statistical Analysis

Statistical analyses were performed in SAS version 9·2 (SAS Institute, Inc.). Categorical variables were compared with χ^2^ or Fisher exact tests, and continuous variables with the Kruskal-Wallis rank-sum test. Odds ratios (OR) with 95% CI were calculated. Exact 95% CIs were computed where applicable. Logistic regression was used for multivariable analysis. The best subset selection method was used to select a final multivariable model from an initial set of variables that had a *p*-value of ≤0·2 on bivariate analysis. Final selection was based on a significance level of 0.05. The best selection method in SAS version 9·2 uses the algorithm of Furnival and Wilson [41] to find the subsets with the highest likelihood score statistic for models with 1, 2, 3, and so on, explanatory variables. We forced relevant variables into the model, and then selected the most parsimonious model with the highest likelihood score statistic. The influence of each two-way interaction on the main effects and other interaction terms, including age, were assessed.

Testing for diarrheagenic *E. coli*, norovirus, sapovirus, or astrovirus was completed for all 107 children who died, and because of resource limitations, a matching set of 107 survivors. Separate from the main cohort study analysis, to examine if these additional pathogens were independently associated with death relative to other etiologies, each child who died was matched to one child who survived on the basis of age in months, identification of nontyphoidal *Salmonella* or *Shigella*, and weight-for-age z-score to control for these factors. A conditional logistic regression model was fit using the approach described above. Matched odds ratios (mORs) with 95% CI were calculated.

Table 1 lists the data collected for each of the subsets and the associated denominators used in the analysis.

**Table 1:** List of the data collected for each of the subsets, and the associated denominators.

Subsets	Purpose	Information Available	Denominators for Analysis	Figures and Tables
All enrolled children^a^	To capture information specifically related to the child's diarrheal episode that led to the hospitalization	Stool testing for nontyphoidal *Salmonella*, *Shigella* species, rotavirus, *Campylobacter* species, *S*. Typhi, and *V. cholerae*Supplemental diarrheal disease questionnaire, which included information on demographics, the child's clinical diarrheal history, and diarrheal treatment during hospitalizationOutcome of the hospitalization	107 children who died1,039 children who survived	Figure 1; Tables 2 and 3
Subset of children with available information from Health and Demographic Surveillance System data^a^	To capture additional clinical information which was captured routinely	Overall clinical course, physical exam by the attending physician, anthropometric measurements, health-seeking behavior, treatments before the hospital visit, and additional laboratory testing	85 children who died788 children who survived	Table 4 and Table 5 b
Matched subset of children who had additional laboratory testing	To carry out testing for diarrheagenic *E. coli*, astrovirus, norovirus, and sapovirus on a subset of children	Stool testing for diarrheagenic *E. coli*, astrovirus, norovirus, and sapovirus on a subset. Due to resource limitations, this was only possible for a small sub-set.	107 children who died107 children who survived (matched to the 107 children who died)	Table 6

a Before initiating analysis data exploration (not shown) was carried out to compare the entire cohort of all enrolled children to the subset of children with available information from Health and Demographic Surveillance System data and no differences were noted.

b For the multivariable logistic regression model, *n* = 84 for children who died and *n* = 781 for children who survived due to missing data.

### Ethical Review

The study protocol was approved by the Institutional Review Board of the US CDC, the KEMRI Scientific Steering Committee (SSC), and the KEMRI National Ethical Review Committee (ERC).

## Results

From May 23, 2005 to May 22, 2007, 1,146 children <5 y old hospitalized with diarrhea were enrolled; 508 (44%) were female. The participation rate was 90%; among those who declined, the reasons were the caretaker was in a hurry (87%), refused (8%), or was unavailable (5%). No caretakers with severely ill children refused participation. Of 1,146 enrolled children (757 and 389 at Siaya District Hospital and Bondo District Hospital, respectively), 107 died during hospitalization, for an in-hospital case fatality ratio (CFR) of 9·3% (9·4% and 9·3% at Siaya District Hospital and Bondo District Hospital, respectively). The sex-specific CFR among children with diarrhea who died during hospitalization was 10·2% for females (52 of 508) and 8·6% for males (55 of 638), *p* = 0·4. The median age at presentation was 9 mo old for children who died and those who survived (Table 2). Eight enrolled children were neonates, of whom none died.

**Table 2:** Demographic and clinical characteristics, enteric pathogens identified, and pathogen-specific CFRs for 1,146 children <5 y old hospitalized with diarrhea, by children who died (*n* = 107) and survived (*n* = 1,039), western Kenya 2005–2007.

Characteristic	Children Who Died (*n* = 107)	Children Who Survived (*n* = 1,039)	Odds Ratio (95% CI)^a^	*p*-Value	Percent CFR (95% CI)
**Demographics, ** ***n*** ** (%) patients**					
Median age, in months (range)	9 (1–59)	9 (<1–59)	—	0·3^b^	—
Infants	62 (58)	676 (65)	0·7 (0·5–1.1)	0·1	—
Female	52 (49)	456 (44)	1·2 (0·8–1·8)	0·4	—
**Clinical characteristics, ** ***n*** ** (%) patients** c					
Bloody diarrhea	10 (9)	86 (8)	1·1 (0·6–2·3)	0·7	—
Watery diarrhea	87 (81)	854 (83)	0·9 (0·6–1·5)	0·8	—
Mucoid diarrhea	82 (78)	889 (86)	0·6 (0·4–1·0)	0·03	—
Vomiting	70 (65)	747 (72)	0·7 (0·5–1·1)	0·2	—
Fever (subjective)	70 (65)	729 (70)	0·8 (0·5–1·2)	0·3	—
Abdominal cramps	70 (67)	626 (61)	1·3 (0·9–2·0)	0·2	—
Nausea	69 (68)	782 (77)	0·6 (0·4–1·0)	0·04	—
Maximum number of stools in 24 h, before hospitalization, median (range)	4 (3–10)	4 (3–10)	—	0·1^b^	—
Median duration of diarrhea before hospitalization, days (IQR)	5 (4–7)	4 (3–5)	—	<0·01^b^	—
Median duration of hospitalization, in days (IQR)	3 (1–5)	3 (1–5)	—	0·2^b^	—
Median duration from illness onset to date of death or discharge, days (IQR)	8 (5–10)	7 (5–10)	—	0·7^b^	—
**Stool testing, ** ***n*** ** (%) patients**					
Whole stool specimen	101 (94)	952 (92)	1·5 (0·7–3·6)	0·3	—
Not cultured	0 (0)	9 (0·9)	0 (0–3·9)	1·0^d^	—
No rotavirus testing	9 (8)	116 (11)	0·7 (0·3–1·5)	0·4	—
Yielded at least 1 pathogen	46 (43)	341 (33)	1·5 (1·0–2·3)	0·03	—
Yielded >1 pathogen	5 (5)	25 (2)	2·0 (0·7–5·3)	0·2	—
**Pathogens identified, ** ***n*** ** (%) patients** e					
Nontyphoidal *Salmonella*	24 (22)	94 (9)	2·9 (1·7–4·8)	<0·01	20·3 (13·1–27·6)^f^
*Shigella* species	12 (11)	30 (3)	4·2 (2·1–8·5)	<0·01	28·6 (14·9–42·2)^f^
Rotavirus	9 (9)	187 (20)	0·4 (0·2–0·8)	<0·01	4·6 (2·4–8·5)^g^
*Campylobacter* species	5 (5)	52 (5)	0·9 (0·4–2·4)	0·9	8·8 (4·8–18·9)^g^
*Salmonella enterica* serotype Typhi	1 (0·9)	2 (0·2)	4·8 (0·1–93·6)	0·3^d^	—h
*V. cholerae*	0 (0)	1 (0·1)	0 (0–182·9)	1·0^d^	—h

a Unadjusted odds ratio from bivariate analysis.

b Kruskal-Wallis test.

c Based on caretakers assessment on admission.

d Fisher exact test, and exact 95% CIs reported.

e Denominators for rotavirus are *n* = 98 for patients who died, and *n* = 923 for patients who survived. Denominators for culturing are *n* = 107 for patients who died, and *n* = 1,030 for patients who survived. 9 enrolled children did not have a culture work up but had viral testing carried out on stool.

f Wald CI for binomial proportion.

g Wilson CI for binomial proportion.

h Not estimated due to insufficient data.

IQR, interquartile range.

Reported symptoms for children who died and those who survived were similar. Overall, bloody diarrhea was recorded in 9% of children who died, and 8% who survived (OR = 1·1; 95% CI 0·6–2·3). Children who died had a significantly longer median duration of diarrhea before hospitalization than children who survived (5 versus 4 d, *p*<0·01), and both groups had similar median durations of hospitalization (3 versus 3 d, *p* = 0·2), and of diarrheal illness (8 versus 7 d, *p* = 0·7) (Table 2).

In total, 92% of specimens tested were whole stool specimens and the remainder were rectal swabs. A higher proportion of children who died than survived had an enteric pathogen identified in their stool (43% versus 33%, OR = 1·5; 95% CI 1·0–2·3) (Table 2).

Children who died were less likely to have rotavirus detected in stool than those who survived (9% versus 20%, OR = 0·4; 95% CI 0·2–0·8). The pathogen-specific CFR for rotavirus was 4·6% (CI 2·4–8·5). The pathogen-specific CFRs were highest for *Shigella* (28·6%, CI 14·9–42·2), and nontyphoidal *Salmonella* (20·3%, CI 13·1–27·6) (Table 2).

**Figure 1:**
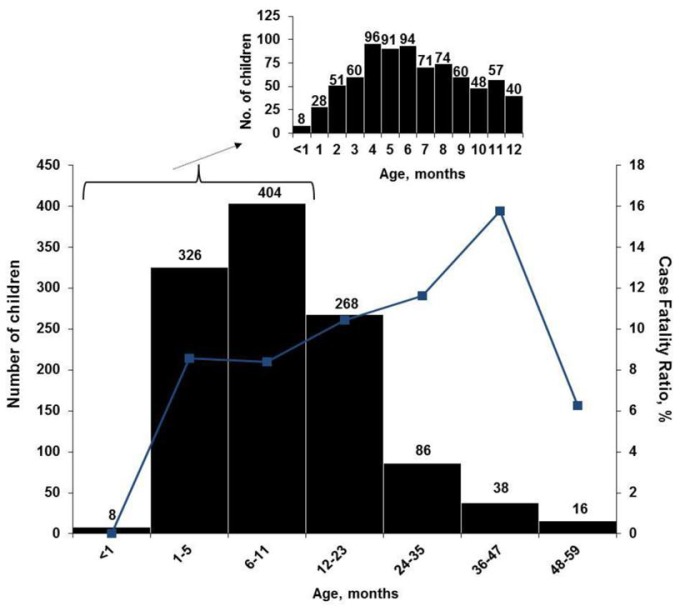
Age distribution (bars) and age-specific CFR (line) of 1,146 children hospitalized with diarrhea, western Kenya 2005–2007. Inset, age distribution by 1-mo periods, for children aged <1 to 12 mo old (*n* = 778).

The highest age-specific all-cause CFRs were among children 2 to 4 y old (Figure 1). When stratified by age groups that were based on developmental stages, breastfeeding and the weaning ages, nontyphoidal *Salmonella* and *Shigella* infections were associated with increased mortality among infants 0–11 mo, and *Shigella* infection was associated with death among children 24–59 mo. No association between any one pathogen examined and increased mortality was seen in children 12–23 mo of age. Although overall rotavirus was most prevalent in infants, those who died were less likely to test positive for rotavirus than survivors. The highest proportion of rotavirus among children who died was in the 24–59-mo-old age category, where 19% had rotavirus identified in their stool. On unadjusted bivariate analysis there was a significant interaction between nontyphoidal *Salmonella* (*p* = 0.007) and age, and rotavirus and age (0.03) (Table 3).

**Table 3:** Select enteric pathogens identified by age group among enrolled children <5 y old hospitalized with diarrhea and had a stool specimen cultured and/or tested for viral pathogens, by children who died (*n* = 107) and survived (*n* = 1,039), western Kenya 2005–2007.

Enteric Pathogen^a^	*n* Children Who Died (%)	*n* Children Who Survived (%)	Odds Ratio (95% CI)^b^	*p*-Value^c^
**Nontyphoidal ** ***Salmonella*** ** species**				
0–11 mo old	18/62 (29)	45/668 (7)	5·7 (3·0–10·6)	0·007
12–23 mo old	4/28 (14)	29/239 (12)	1·2 (0·4–3·7)	
24–59 mo old	2/17 (12)	20/123 (16)	0·7 (0·1–3·2)	
***Shigella*** ** species**				
0–11 mo old	4/62 (6)	11/668 (2)	4·1 (1·3–13·3)	0·4
12–23 mo old	2/28 (7)	10/239 (4)	1·8 (0·4–8·5)	
24–59 mo old	6/17 (35)	9/123 (7)	6·9 (2·1–23·0)	
**Rotavirus**				
0–11 mo old	5/56 (9)	155/606 (26)	0·3 (0·1–0·7)	0·03
12–23 mo old	1/26 (4)	23/203 (11)	0·3 (0·04–2·4)	
24–59 mo old	3/16 (19)	9/114 (8)	2·7 (0·6–11·2)	

a Denominators for rotavirus are *n* = 98 for patients who died, and *n* = 923 for patients who survived. Denominators for culturing are *n* = 107 for patients who died, and *n* = 1,030 for patients who survived. 9 enrolled children did not have a culture work up but had viral testing carried out on stool.

b Unadjusted odds ratio from bivariate analysis (odds of death among those with the enteric pathogen versus those without the pathogen within specific age groups).

c *p*-Value for the interaction term.

Sub-typing results are shown in Table S1. Serotyping of the nontyphoidal *Salmonella* isolates revealed that 75% of the isolates among children who died and 70% among those who survived where *S*. Typhimurium. *Shigella* species distribution among children who died versus those who survived was somewhat different. For *Campylobacter* isolations, 80% of isolates from children who died and 83% from survivors were *C. jejuni*. Rotavirus genotyping results were available for four (44%) of the nine children who died with rotavirus; three were genotype G2P[4] and one was P[6] (Table S1).

We observed no significant differences between survivors and decedents in the proportion of nontyphoidal *Salmonella* or *Shigella* isolates that were resistant to any of the antimicrobial agents tested.

### Subset of Children with Available Information from HDSS

Risk factors for death identified from the bivariate analysis of a subset of 85 (79%) children who died and 788 (76%) children who survived and had HDSS information available included the following: having a clinical diagnosis of malnutrition on admission or at discharge/death; being malnourished as assessed via anthropometry; oral thrush on physical exam; a clinical diagnosis of dehydration on admission, physical exam, or discharge/death; returning to the hospital for further treatment or being referred for further treatment from another health facility; and being taken to the hospital by a relative who was not the child's biological parent. Being awake and interactive or irritable on physical exam was protective against death (Table 4).

**Table 4:** Clinical information for children <5 y old hospitalized with diarrhea in rural western Kenya (HDSS subset: 85 children who died, 788 children who survived).

Characteristic	*n* Children Who Died (%)	*n* Children Who Survived (%)	Odds Ratio (95% CI)^a^	*p*-Value
**Clinical diagnosis on admission**				
Cerebral malaria	0/85 (0)	1/788 (0·1)	0 (0–176·1)	1·0^b^
Convulsions	1/85 (1)	10/788 (1)	0·9 (0·02–6·7)	1·0^b^
Dehydration	44/85 (52)	302/788 (38)	1·7 (1·1–2·7)	0·02
Gastroenteritis	38/85 (45)	380/788 (48)	0·9 (0·6–1·4)	0·5
Malaria	59/85 (69)	582/788 (74)	0·8 (0·5–1·3)	0·4
Malnutrition	19/85 (22)	46/788 (6)	4·6 (2·6–8·4)	<0·01
Pneumonia	21/85 (25)	166/788 (21)	1·3 (0·7–2·1)	0·4
Severe anemia	8/85 (9)	68/788 (9)	1·1 (0·5–2·4)	0·8
URTI	4/85 (5)	40/788 (5)	0·9 (0·2–2·7)	1·0^b^
Wound/physical injury	0/85 (0)	1/788 (0·1)	0 (0–176·1)	1·0^b^
Other admission diagnosis^c^	15/85 (18)	114/788 (14)	1·2 (0·7–2·3)	0·4
**Physical exam findings**				
Convulsions during admission	3/85 (4)	10/787 (1)	2·9 (0·5–11·6)	0·1^b^
Cough during interview	21/85 (25)	159/788 (20)	1·3 (0·8–2·2)	0·3
Grunting	26/85 (31)	201/788 (26)	1·3 (0·8–2·1)	0·3
Dehydration^d^	58/85 (68)	404/788 (51)	2·0 (1·3–3·3)	<0·01
Jaundice	5/85 (6)	33/788 (4)	1·4 (0·4–3·8)	0·4^b^
Nasal flaring	39/85 (46)	402/788 (51)	0·8 (0·5–1·3)	0·4
Enlarged lymph nodes	0/85 (0)	3/788 (0·4)	0 (0–16·0)	1·0^b^
Runny nose	3/85 (4)	44/788 (6)	0·6 (0·1–2·0)	0·6^b^
Bipedal edema	6/85 (7)	22/788 (3)	2·6 (1·0–6·7)	0·03
Palpable liver	1/85 (1)	11/788 (1)	0·8 (0·02–5·9)	1·0^b^
Red eyes	0/85 (0)	3/788 (0·4)	0 (0–16·0)	1·0^b^
Pulling at ribs	40/85 (47)	341/788 (43)	1·2 (0·7–1·8)	0·5
Palpable spleen	2/85 (2)	37/788 (5)	0·5 (0·06–2·0)	0·4^b^
Temperature above 37·5°C	33/85 (39)	387/788 (49)	0·7 (0·4–1·0)	0·07
Oral thrush	41/85 (48)	186/788 (24)	3·0 (1·9–4·8)	<0·01
Fast breathing^e^	29/84 (35)	346/788 (44)	0·7 (0·4–1·1)	0·1
Evaluation of mental status^f^				
Awake and interactive or irritable	5/84 (6)	145/788 (18)	0·3 (0·1–0·7)	<0·01
Lethargic/comatose	79/84 (94)	643/788 (82)	Referent	
**Anthropometry**				
Weight-for-length/height z-score <−2	30/72 (42)	184/742 (25)	2·2 (1·3–3·6)	<0·01
Length/height-for-age z-score <−2	38/72 (53)	295/742 (40)	1·7 (1·0–2·8)	0·03
Weight-for-age z-score <−2	46/72 (64)	297/742 (40)	2·4 (1·5–3·8)	<0·01
**Laboratory findings**				
*P. falciparum* (on blood smear)	11/85 (13)	204/788 (26)	0·4 (0·2–0·8)	<0·01
Median parasite density (per µl)	5,041 (80–179,196)	19,549 (27–197,651)	—	0·2^g^
High parasite density^h^	3/85 (4)	100/788 (13)	0·3 (0·05–0·8)	0·01^b^
Median hemoglobin (g/dl)	9·2 (2·7–13·4)	9·5 (1·4–17·6)	—	0·3^g^
Severe anemia^i^	9/85 (11)	76/788 (7)	1·6 (0·8–3·4)	0·2
**Clinical diagnosis on discharge/death**				
Cerebral malaria	0/85 (0)	2/788 (0·3)	0 (0–32·3)	1·0^b^
Convulsions	1/85 (1)	4/788 (0·5)	2·3 (0·04–23·9)	0·4^b^
Dehydration	40/85 (47)	184/788 (23)	2·9 (1·8–4·6)	<0·01
Gastroenteritis	32/85 (38)	297/788 (38)	0·9 (0·6–1·6)	1·0
Malaria	57/85 (67)	526/788 (67)	1·0 (0·6–1·6)	1·0
Malnutrition	15/85 (18)	39/788 (5)	4·1 (2·2–7·8)	<0·01
Pneumonia	23/85 (27)	181/788 (23)	1·2 (0·7–2·1)	0·4
Severe anemia	7/85 (8)	44/788 (6)	1·4 (0·7–3·0)	0·3
URTI	2/85 (2)	22/788 (3)	0·8 (0·1–3·5)	1·0^b^
Wound/physical injury	0/85 (0)	1/788 (0·1)	0 (0–176·1)	1·0^b^
Other diagnosis on discharge/death^c^	13/85 (15)	124/788 (16)	1·0 (0·5–1·8)	0·9
**Other diagnosis**				
HIV/AIDS^j^	2/85 (2·4)	5/788 (0·6)	3·8 (0·4–23·4)	0·14^b^
**Admitted after 5 pm**	30/84 (36)	279/787 (35)	1·0 (0·6–1·6)	1·0
**Returning for further treatment/referred**	15/84 (18)	76/788 (10)	2·0 (1·1–3·7)	0·02
**Taken to the hospital by a relative (not parent)**	7/84 (8)	23/787 (3)	3·0 (1·3–7·3)	0·01

a Unadjusted odds ratio from bivariate analysis.

b Fisher exact test, and exact 95% CIs reported.

c Where available included conjunctivitis, neonatal sepsis, meningitis, respiratory tract infection, pulmonary tuberculosis, sickle-cell disease.

d Dehydration on physical exam defined as sunken eyes, loss of skin turgor (slow skin pinch return [≤2 s] or very slow return [>2 s]) or sunken fontenelle.

e Fast breathing defined as ≥60 breaths per minute for children 0–<2 mo old, ≥50 breaths per minute for children 2–11 mo, and ≥40 breaths per minute for children 12–59 mo.

f Lethargic and comatose were combined as being in a state of coma was rarely reported (*n* = 2 for children who died, *n* = 3 for children who survived).

g Kruskal-Wallis test.

h Cut-off based on the overall median parasite density (19,149 µl), high parasite density was defined as ≥20,000 parasites per µl.

i Severe anemia defined as a hemoglobin concentration <5 g/dl.

j Diagnoses considered to indicate HIV/AIDS were a diagnosis of immune suppression syndrome, or if a child was documented as being on antiretroviral therapy on their medical records. Classified separately due to non-routine data collection.

Having a clinical diagnosis of malaria, or having *Plasmodium falciparum* parasites on blood smear, and having a high malaria parasite density were not associated with increased risk of death (Table 4).

While a higher proportion of enrolled children who died than survived were diagnosed with HIV/AIDS (2·4% versus 0·6%, *p* = 0·14), the difference was not statistically significant (Table 4).

Previously seeking care for the current diarrheal illness was associated with death. No specific pre-hospitalization treatment or in-hospital treatment for the current hospitalization was identified as a risk or protective factor for death during the child's hospitalization (Table S2).

Twelve main-effect variables were selected for the final multivariable logistic regression model. Given the significant interaction of nontyphoidal *Salmonella* and age observed in the unadjusted bivariate analysis, the interaction term of nontyphoidal *Salmonella* and age was included in the model. Of the main effects, six were independently associated with an increased odds of death among enrolled children during hospitalization: nontyphoidal *Salmonella* species isolated from the stool of infants (adjusted OR [aOR] = 6·8; 95% CI 3·1–14·9), *Shigella* species isolated from stool (aOR = 5·5; 95% CI 2·2–14·0), having a clinical diagnosis of malnutrition on admission (aOR = 4·2; 95% CI 2·1–8·7), having a diagnosis of dehydration on discharge or death (aOR = 2·5; 95% CI 1·5–4·1), having oral thrush on physical exam (aOR = 2·3; 95% CI 1·4–3·8), and having previously sought care at a hospital for the current diarrheal illness (aOR = 2·2; 95% CI 1·2–3·8) (Table 5). Being awake and interactive or irritable as opposed to being lethargic or in a coma was associated with a reduced odds of death in the model (aOR = 0·3; 95% CI 0·1–0·9). We assessed all pairwise interactions, and other main effects, and none reached the 0.05 level of significance in the multivariable analysis.

**Table 5:** Factors independently associated with an increased or decreased risk of death among children <5 y old hospitalized with diarrhea in a multivariable logistic regression analysis, western Kenya 2005–2007.

Factor^a^	aOR^b^	(95% CI)^c^	*p*-Value
**Nontyphoidal ** ***Salmonella*** ** species isolated from stool**			
0–11 mo old	6·8	(3·1–14·9)	0.06^d^
12–23 mo old	2·1	(0·5–8·5)	
24–59 mo old	0·5	(0·05–5·4)	
***Shigella*** ** species isolated from stool**	5·5	(2·2–14·0)	<0·01
**Clinical diagnosis of malnutrition on admission**	4·2	(2·1–8·7)	<0·01
**Dehydration diagnosed on discharge/death**	2·5	(1·5–4·1)	<0·01
**Oral thrush on physical exam**	2·3	(1·4–3·8)	<0·01
**Previously sought care at a hospital for the current illness**	2·2	(1·2–3·8)	<0·01
**Awake and interactive or irritable on physical exam**	0·3	(0·1–0·9)	0·02

The denominators for the data included in this analysis were *n* = 84 children who died and *n* = 781 children who survived, i.e. children for whom additional HDSS inpatient data were available.

a Twelve variables were included in the final model with results shown for the six main effects, and the interaction between nontyphoidal *Salmonella* and age. The five additional non-significant variables were gender, duration of diarrhea, a diagnosis of HIV/AIDS, seeking care at a traditional healer previous to hospital visit, and referred for care.

b aOR, whereby all odds ratios control for other factors in the model.

c Confidence interval.

d *p*-Value for the interaction term of nontyphoidal *Salmonella* and age.

### Matched Subset of Children Who Had Additional Laboratory Testing

In the matched conditional logistic regression analysis of 107 children who died and 107 matched survivors, a higher proportion of children who survived than died had enteroaggregative *E. coli* (EAEC) (36% versus 30%), atypical EPEC (19% versus 9%), typical EPEC (19% versus 8%), enterotoxigenic *E. coli* (ETEC) (10% versus 6%), norovirus (9% versus 4%,), and sapovirus (8% versus 2%) identified. The same proportion of children who died and survived tested positive for astrovirus (7% versus 7%). No shiga toxin-producing *E. coli* (STEC) or enteroinvasive *E. coli* (EIEC) was identified. In the model, diarrheagenic *E. coli*, norovirus, astrovirus, and sapovirus were not found significantly more in decedents compared with survivors (Table 6). An unmatched analysis is also shown in Table 6 and provided similar results. No significant interactions with age and the enteric pathogens assessed in the subset were identified.

**Table 6:** Matched conditional logistic regression analysis of pathogen-specific risk factors for death among children <5 y old who died during hospitalization with diarrhea (*n* = 107), compared with children <5 y old with diarrhea who survived hospitalization (*n* = 107), western Kenya 2005–2007.

Enteric Pathogen	*n* Children Who Died (%)	*n* Children Who Survived (%)	Unmatched Analysis		Matched Analysis			
			OR (95% CI)^a^	aOR (95% CI)^b^	mOR (95% CI)^c^	*p*-Value for mOR	aOR (95% CI)^d^	*p-*Value for aOR
**Diarrheagenic ** ***E. coli***								
Enteroaggregative *E. coli*	31/104 (30)	38/107 (36)	0·6 (0·3–1·1)	0·7 (0·3–1·5)	0·8 (0·4–1·4)	0·41		
Atypical enteropathogenic *E. coli* e	9/104 (9)	20/107 (19)	0·2 (0·06–0·6)	0·3 (0·08–1·1)	0·4 (0·2–1·0)	0·05	0·3 (0·6–1·1)	0·07
Typical enteropathogenic *E. coli* f	8/104 (8)	20/107 (19)	0·3 (0·1–0·8)	0·3 (0·1–0·9)	0·4 (0·2–0·9)	0·02	0·4 (0·1–1·1)	0·08
Enterotoxigenic *E. coli*	6/104 (6)	11/107 (10)	0·6 (0·2–2·0)		0·6 (0·2–1·5)	0·25		
Shiga toxin-producing *E. coli*	0/104 (0)	0/107 (0)						
Enteroinvasive *E. coli*	0/104 (0)	0/107 (0)						
**Viruses**								
Astrovirus	7/95 (7)	7/98 (7)	1·1 (0·3–3·7)		1·1 (0·40–3·3)	0·88		
Norovirus	4/95 (4)	9/98 (9)	0·5 (0·1–2·1)		0·5 (0·10–1·5)	0·20		
Sapovirus	2/94 (2)	8/98 (8)	0·1 (0·01–0·8)	0·1 (0·01–1·0)	0·2 (0·04–1·1)	0·06	0·2 (0·02–1·5)	0·10

a Unmatched, unadjusted odds ratio.

b Unmatched aOR, whereby, odds ratios control for age and the significant variables from the model shown in Table 5.

c Matched odds ratio on univariate analysis.

d aOR, whereby, matched odds ratios control the significant variables from the model shown in Table 5.

e Represents bfpA− EPEC.

f Represents bfpA+ EPEC.

mOR, matched odds ratio.

## Discussion

Although diarrhea is a major cause of mortality in young children in developing countries, few studies comprehensively examine infectious diarrheal etiologies associated with death [13]. Our study is unique because it examines risk factors for childhood diarrheal mortality including a range of diarrheal etiologies in a setting of a high infant and child mortality, and high malaria and HIV prevalence. Rotavirus was the most common etiology of diarrhea in hospitalized children in this rural area but was not the most frequently identified pathogen among in-hospital fatalities. The pathogen-specific CFR for rotavirus was lower than for other enteric pathogens, particularly nontyphoidal *Salmonella* and *Shigella* species. Children who died were more likely to have had nontyphoidal *Salmonella*, or *Shigella* infections than children who survived.

The children who died while hospitalized for diarrhea were vulnerable for several reasons. They had a significantly longer duration of diarrhea before reaching the hospital, and were more likely to be returning to the hospital, a marker for severe illness, or inadequate treatment/premature discharge on previous admission, as observed in previous studies [16],[17]. The median age of death was 9 mo old. Those with nontyphoidal *Salmonella* infections, which were associated with 22% of all deaths, were particularly young (median age 7 mo). This young age coincides with the critical weaning period when foods are introduced and *Salmonella*-specific maternal antibody is lost with consequent elevated risk of diarrhea [42]–[44]. Children who died had other co-morbidities, which were identified as independent risk factors for death, such as malnutrition, oral thrush (which can be associated with HIV/AIDS infection), and dehydration, as has been previously documented [14],[15],[18]. In general, all children in this study had substantially more stunting and wasting and a higher proportion were underweight than children in communities in Nyanza Province [45]. Of note, a lower or similar prevalence of a diarrheal pathogen among children who died compared to survivors does not necessarily indicate that infection did not contribute to mortality, but that the CFRs were lower for such pathogens than for others.

Most cases of non-bloody, non-septic bacterial diarrhea do not require antimicrobial therapy and resolve with symptomatic support (e.g., oral rehydration); however, >75% of enrolled children were treated in-hospital with an antimicrobial drug. It has been demonstrated in a previous study [46] and in the current study (unpublished data) that the utility of the commonly available antimicrobials for treating bacterial diarrhea in this area is substantially limited by reduced antimicrobial susceptibility, particularly for *Shigella* and nontyphoidal *Salmonella*. Training and oversight on judicious use of antimicrobial drugs, and enhanced access to laboratory diagnostics for diarrheal diseases, including capacity for blood culture, are warranted to appropriately treat potentially fatal diarrhea.

The study hospitals intermittently ran out of stocks of many critical and life-saving supplies for the treatment of diarrhea, such as ORS, and pediatric intravenous (IV) fluids, needles, or tubing during the study. In addition, in a study assessing community availability of ORS carried out in the area during the same time period, there was a documented lack of widespread availability of ORS packets with only 4% of shops and 48% of pharmacies in the area having ORS available for sale, resulting in very limited community access to life-saving treatment for dehydration outside of the health facility [47],[48].

This study was subject to several important limitations. It only captured in-hospital childhood deaths, and likely missed a substantial number of additional deaths that occurred at home. In resource-limited settings there are inherent biases in studying the etiology of diarrheal deaths in hospitals because rotavirus can be successfully treated with hydration relative to bacterial agents, which may require effective antimicrobial therapy, necessitating knowledge of the causative agent and its antimicrobial susceptibility, which is often not feasible in such settings. Since most diarrheal deaths occur at home where rehydration is less accessible, the etiologic picture of overall childhood diarrheal deaths could be different if community deaths were assessed. HIV counseling and testing were not routinely offered at the time of the study; therefore HIV testing results are not available for participants. Where available, we relied on HIV diagnosis based on clinical features, which may be subject to biases in assessing the factors contributing to diarrheal disease among participants since HIV infection at early stages may have been missed and not all data were routinely captured. Also, given the number of infectious enteric pathogens and clinical factors assessed our model may not have been able to differentiate between clinical factors that had similar effect sizes. The study did not capture other potentially relevant information, such as whether illness was associated with bacteremia (blood cultures were not done), breastfeeding status, and did not specifically ask about pre-hospitalization ORS use. Assessing the prognostic performance of the factors associated with mortality in the detection of patients at high risk of death, as has been carried out in previous mortality studies [49], would be important in future analysis, as would expanding the testing panel to include *Cryptosporidium*, *Giardia*, and other enteric agents. With regard to the sensitivity of the tests used, culture is the gold standard for the detection of bacterial agents, and the limit of detection of the viral RT-PCR assays ranges from 10–100 viral particles/reaction.

Since vaccines for most bacterial diarrheal diseases are in the distant future, and roll-out of rotavirus vaccines worldwide is as yet limited, expedited implementation of the new Kenyan Ministry of Public Health and Sanitation (MoPHS) policy on the control and management of diarrheal diseases in children <5 y old is critical [50]. The strategy focuses on home-based case management, including promotion of ORS and zinc use, prompt and effective health facility-based case management, diarrhea prevention through improved nutrition, water, sanitation, and hygiene, and the introduction of rotavirus vaccine, behavior change communication, and logistics management.

The national supply chain management of critical diarrhea treatment supplies such as ORS, pediatric IV fluids, and zinc, should be strengthened, and enforced systematic inventory monitoring of these supplies should take place at health facilities. The implementation of an improved supply chain, which is contained in the new MoPHS policy [50], will help improve the quality of inpatient pediatric care and prevent unnecessary diarrheal deaths.

The findings of particular clinical relevance are that immediate priority should be given to the management of children presenting to the hospital with diarrhea who are at high risk of death, including those who have previously sought care at a health facility for their illness, are dehydrated, have oral thrush, and are malnourished. In addition to receiving appropriate diarrhea case management, malnourished children with diarrhea should be provided nutritional rehabilitation. Further to identifying children at high risk for death from diarrhea in the hospital, this study can help inform policy makers on priority areas for interventions to reduce childhood diarrhea requiring hospitalization or resulting in death, such as the use of zinc for diarrhea management, reemphasis on community level promotion of ORS, water, sanitation and hygiene interventions, and the development and roll-out of new enteric vaccines.

## Supporting Information

Table S1 **Laboratory characterization of the enteric pathogens identified from children hospitalized with diarrhea, western Kenya 2005–2007.**
(DOCX)

Table S2 **Health-seeking behavior and treatments sought before hospitalization and treatments administered during hospitalization with diarrhea.**
(DOCX)

## Abbreviations

aOR: adjusted odds ratio
CDC: US Centers for Disease Control and Prevention
CFR: case fatality ratio
EPEC: enteropathogenic *E. coli*
HDSS: Health and Demographic Surveillance System
KEMRI: Kenya Medical Research Institute
OR: odds ratio
ORS: oral rehydration solution
WHO: World Health Organization
