# Supplementary material for: Risk Factors for Death among Children Less than 5 Years Old Hospitalized with Diarrhea in Rural Western Kenya, 2005–2007: A Cohort Study
Source: PLoS Med. 2012 Jul 3;9(7):e1001256. doi: 10.1371/journal.pmed.1001256 (PMC3389023; doi:10.1371/journal.pmed.1001256)
Supplement: Table S1 — Laboratory characterization of the enteric pathogens identified from children hospitalized with diarrhea, western Kenya 2005–2007. (DOCX) [file pmed.1001256.s001.docx]

**SUPPORTING INFORMATION**

**TABLE S1. Laboratory characterization of the enteric pathogens identified from children hospitalized with diarrhea, western Kenya 2005-2007.**

| **Enteric pathogen** | **Children who died**  **n (%)** | **Children who survived**‡  **n (%)** |
| --- | --- | --- |
| **Nontyphoidal *Salmonella*, serotype** | **(n=24)** | **(n=87)*** |
| *Salmonella enterica* serotype Typhimurium | 18 (75) | 61 (70) |
| *Salmonella enterica* serotype Enteritidis | 5 (21) | 12 (14) |
| *Salmonella enterica* serotype IIIb 48:i:z | 1 (4) | 0 (0) |
| *Salmonella enterica* serotype Heidelberg | 0 (0) | 2 (2) |
| *Salmonella enterica* serotype Newport | 0 (0) | 2 (2) |
| *Salmonella enterica* serotype Chailey | 0 (0) | 1 (1) |
| *Salmonella enterica* serotype Haifa | 0 (0) | 1 (1) |
| *Salmonella enterica* serotype Infantis | 0 (0) | 1 (1) |
| *Salmonella enterica* serotype Ituri | 0 (0) | 1 (1) |
| *Salmonella enterica* serotype Mowanjum | 0 (0) | 1 (1) |
| *Salmonella enterica* serotype Saintpaul | 0 (0) | 1 (1) |
| *Salmonella enterica* serotype Virchow | 0 (0) | 1 (1) |
| *Salmonella enterica* serotype Guildford | 0 (0) | 1 (1) |
| *Salmonella enterica* serotype Aberdeen | 0 (0) | 1 (1) |
| *Salmonella enterica* serotype Zanzibar | 0 (0) | 1 (1) |
|  |  |  |
| ***Shigella*, species** | **(n=12)** | **(n=30**) |
| *Shigella dysenteriae* Type 2 | 2 (17) | 2 (7) |
| *Shigella flexneri* 2a | 2 (17) | 3 (10) |
| *Shigella boydii* Type 2 | 2 (17) | 0 (0) |
| *Shigella flexneri* 1b | 1 (8) | 4 (13) |
| *Shigella flexneri* 2b | 1 (8) | 10 (33) |
| *Shigella flexneri* 3a | 1 (8) | 0 (0) |
| *Shigella flexneri* 3b | 1 (8) | 1 (3) |
| *Shigella flexneri* 4a | 1 (8) | 3 (10) |
| *Shigella sonnei* (phase I) | 1 (8) | 0 (0) |
| *Shigella flexneri* 6 | 0 (0) | 2 (7) |
| *Shigella sonnei* (phase II) | 0 (0) | 5 (17) |
|  |  |  |
| ***Campylobacter*, species** | **(n=5)** | **(n=52)** |
| *Campylobacter jejuni* | 4 (80) | 43 (83) |
| *Campylobacter coli* | 1 (20) | 6 (12) |
| *Campylobacter* species | 0 (0) | 3 (6) |
|  |  |  |
| ***Vibrio*, species** | **(n=0)** | **(n=1)** |
| *Vibrio cholerae*, nontoxigenic, non-O1, non-O139 | 0 (0) | 1 (100) |
|  |  |  |
| **Rotavirus, genotype**† | **(n=4)** |  |
| G2P[4] | 3 (75) | -- |
| P[6] | 1 (25) | -- |

**NOTE**: *87 (93%) of the 94 *Salmonella* isolates from children who survived were available for serotyping.

†The results of rotavirus genotyping were available for 4 (44%) of the 9 children who died with rotavirus identified in their stool. Results are being finalized from a separate analysis specifically examining rotavirus genotyping which included a proportion of the children enrolled in this study (CDC unpublished data).

‡No significant differences were observed between the children who died and survived for any of the enteric pathogens characterized.
