# Supplementary material for: Risk Factors for Death among Children Less than 5 Years Old Hospitalized with Diarrhea in Rural Western Kenya, 2005–2007: A Cohort Study
Source: PLoS Med. 2012 Jul 3;9(7):e1001256. doi: 10.1371/journal.pmed.1001256 (PMC3389023; doi:10.1371/journal.pmed.1001256)
Supplement: Table S2 — Health-seeking behavior and treatments sought before hospitalization and treatments administered during hospitalization with diarrhea. (DOCX) [file pmed.1001256.s002.docx]

**SUPPORTING INFORMATION**

**TABLE S2. Health seeking behavior and treatments sought before hospitalization and treatments administered during hospitalization with diarrhea.**

| **Characteristic** | **Children who**  **died**  **n (%)** | **Children who**  **survived**  **n (%)** | **Odds Ratio**  **(95% CI)**§ | **p-value** |
| --- | --- | --- | --- | --- |
| *Pre-Hospitalization Care Seeking* | *(n=84)* | *(n=787)* |  |  |
| Previously sought care for current illness | 75 (89) | 614 (78) | 2·3 (1·2–4·8) | 0·02 |
|  | *(n=75)* | *(n=614)* |  |  |
| Hospital | 25 (33) | 135 (22) | 1·8 (1·1–3·0) | 0·03 |
| Pharmacy | 14 (19) | 167 (27) | 0·6 (0·3–1·1) | 0·1 |
| Health centre | 11 (15) | 80 (13) | 1·1 (0·6–2·3) | 0·7 |
| Private clinic | 11 (15) | 64 (10) | 1·5 (0·7–2·9) | 0·3 |
| Traditional healer | 5 (7) | 18 (3) | 2·4 (0·9–6·6) | 0·09 |
| Family friend | 1 (1) | 25 (4) | 0·3 (0·04–2·4) | 0·3* |
| Shop | 0 (0) | 28 (5) | 0 (0–0·9) | 0·06* |
| Nyamrerwa‡ | 0 (0) | 22 (4) | 0 (0–1·2) | 0·2* |
| Community health worker | 0 (0) | 2 (0·3) | 0 (0–28·5) | 1·0* |
| Bush doctor | 0 (0) | 1 (0·2) | 0 (0–155·5) | 1·0* |
| Other location | 18 (24) | 136 (22) | 1·1 (0·6–1·9) | 0·7 |
|  |  |  |  |  |
| *Pre-Hospitalization Treatments* | *(n=70)* | *(n=612)* |  |  |
| Traditional medicine | 11 (16) | 73 (12) | 1·4 (0·7–2·7) | 0·4 |
| Any antibiotic | 28 (40) | 210 (34) | 1·3 (0·8–2·1) | 0·3 |
| Anti-helminthics | 10 (14) | 97 (16) | 0·9 (0·4–1·8) | 0·7 |
| Sulfadoxine-pyramethamine | 5 (7) | 52 (9) | 0·8 (0·3–2·1) | 0·7 |
| Analgesics (panadol, asprin) | 29 (41) | 324 (53) | 0·6 (0·4–1·0) | 0·07 |
| Chloroquine | 2 (3) | 12 (2) | 1·5 (0·2–6·8) | 0·6* |
| Unknown injection | 8 (11) | 50 (8) | 1·5 (0·7–3·2) | 0·4 |
| Amodiaquine | 10 (14) | 98 (16) | 0·9 (0·4–1·8) | 0·7 |
| Quinine | 4 (6) | 64 (10) | 0·5 (0·1–1·5) | 0·3* |
| Other treatment | 33 (47) | 251 (41) | 1·3 (0·8–2·1) | 0·3 |
|  |  |  |  |  |
| *In-Hospital Treatments* | *(n=107)* | *(n=1,039)* |  |  |
| Any antibiotic | 81 (76) | 792 (76) | 1·0 (0·6–1·5) | 0·9 |
| Anti-helminthics | 5 (5) | 38 (4) | 1·3 (0·5–3·4) | 0·6 |
| Analgesics | 38 (36) | 450 (43) | 0·7 (0·5–1·1) | 0·1 |
| Antimalarials | 65 (61) | 723 (70) | 0·7 (0·4–1·0) | 0·06 |
| Anti-convulsant | 1 (1) | 12 (1) | 0·8 (0·02–5·6) | 1·0* |
| Intravenous fluids† | 93 (91) | 869 (88) | 1·5 (0·7–3·0) | 0·3 |
| Oral rehydration salts† | 83 (81) | 750 (76) | 1·4 (0·8–2·4) | 0·2 |
| Intravenous fluids and oral rehydration salts† | 79 (77) | 667 (67) | 1·7 (1·0–2·7) | 0·04 |
| Other treatment | 50 (47) | 457 (44) | 1·1 (0·8–1·7) | 0·6 |

**NOTE**: *Fisher’s exact test, and exact 95% CIs reported.

†Denominator for children who died is n=102 and for children who survived is n=1,000.

‡Village health workers/birth attendants who live in the community and have either inherited skills in traditional medicine, or have had some basic health care training provided by a non-governmental organization.

§Unadjusted Odds Ratio from bivariate analysis, CI denotes confidence interval.
